# Absence of a positive correlation between CRP and leptin in rheumatoid arthritis

**DOI:** 10.1016/j.heliyon.2016.e00205

**Published:** 2016-12-05

**Authors:** Seyed Reza Najafizadeh, Ghasem Farahmand, Ahmad Tahamoli Roudsari, Behnam Heidari, Mehrdad Larry, Arash Aghajani Nargesi, Atieh Pajouhi, Alireza Esteghamati, Manouchehr Nakhjavani

**Affiliations:** a Rheumatology Research Center, Vali-Asr Hospital, Tehran University of Medical Sciences, Tehran, Iran; b Endocrinology and Metabolism Research Center (EMRC), Vali-Asr Hospital, Tehran University of Medical Sciences, Tehran, Iran; c Hamedan University of Medical Sciences, Hamedan, Iran

**Keywords:** Medicine, Internal medicine, Immunology, Endocrinology

## Abstract

**Aims:**

Rheumatoid Arthritis (RA) is a model of chronic inflammatory disease. In this study we evaluated the correlation of leptin and CRP in patients with RA and normal controls.

**Main methods:**

A total of 75 patients with RA and 40 healthy adults were recruited in this case-control study. RA patients were categorized into high (DAS–28 > 3.2) and low activity (DAS ≤ 3.2) group according to their DAS-28 score.

**Key findings:**

Leptin level was significantly correlated with CRP in healthy controls (r = 0.365; p < 0.05), but this correlation was lost in RA patients (r = 0.095, p = 0.41). Patients with RA had higher serum leptin levels compared to healthy controls (P < 0.01). No difference in serum leptin level was observed between patients with high and low activity disease. Also leptin was correlated with BMI in healthy controls (r = 0.326, p = 0.037). This correlation was not present in RA patients (r = 0.039, p = 0.756).

**Significance:**

We observed that the physiologic correlation between leptin and CRP and BMI and CRP was not present RA patients. This is a new study reporting the lost correlation between leptin and CRP in RA patients.

## 1 Introduction

The role of cytokines in development and progression of rheumatoid arthritis has been studied earlier [1, 2, 3, 4]. Leptin is a non-glycosylated peptide hormone belonging to type 1 cytokine superfamily, and its structure is similar to interleukin (IL)-2, IL-6 and granulocyte colony-stimulating factor (G-CSF) [5, 6]. It is mainly produced by white adipose tissue and is known as the regulator of appetite and energy intake, alongside hematopoiesis, angiogenesis, endocrine and more recently as a mediator of immune mediated and inflammatory processes [7]. In addition to adipose tissue and inflammation, catecholamine, smoking, hormonal factors can affect leptin secretion [8]. It is shown that leptin plays role in the activation and proliferation of various immune cells and cytokine production that potentially affects both innate and adaptive immune systems and also inflammation in human and animal models [7, 9, 10]. The proinflammatory role of leptin in immune-mediated conditions such as SLE, psoriasis, MS and RA has been reported [10]. Regarding the inflammatory properties of leptin, determining its correlation with RA and its activity has been studied [3, 4, 11, 12, 13].

We previously showed the impact of inflammation on altering the physiological and pathological correlations [14, 15, 16, 17]. It is previously shown that leptin and CRP are correlated in healthy individuals [15, 18], and we reported the lost correlation between CRP and leptin in type 2 diabetes mellitus [15]. We were interested in studying this correlation in RA as a model of chronic inflammatory disease. We assume that the inflammation may have a role in disappearing of this correlation. To our knowledge this is a new study comparing this relationship in RA patients and healthy subjects.

## 2 Methods

### 2.1 Study design and subject selection

The present case-control study was conducted from April 2013 to March 2014 in Imam Khomeini hospital of the Tehran University of Medical Sciences, Tehran, Iran. 75 RA patients were recruited from rheumatology clinic of Vali-Asr hospital affiliated with the Tehran University of Medical Sciences. In addition, 40 healthy adults were selected from diabetes clinic of Vali-Asr hospital. The diagnosis of RA was made according to the 2010 American College of Rheumatology (ACR 2010) guidelines by an expert rheumatologist [19]. According to the DAS-28 criteria, 40 patients with high activity RA and 35 patients with low activity RA were included in the study. Low activity RA was defined as DAS-28 ≤ 3.2 and high activity RA was defined as DAS–28 > 3.2 [20]. Age and sex were matched between cases and controls. Exclusion criteria were malnutrition, malignancies, chronic diseases other than RA, pregnancy, lactation, taking prednisolone > 7.5 mg/day, taking methotrexate > 15 mg/week, and taking immunosuppressive drugs other than methotrexate and prednisolone.

Research was performed according to the Declaration of Helsinki principles and was approved by the ethics committee of the Tehran University of Medical Sciences. All participants gave written informed consent before enrollment.

### 2.2 Clinical and laboratory measurements

Demographic and anthropometric data including age, sex, height, weight drug history, and disease duration were recorded. Body mass index (BMI) was calculated according to the Quetelet formula. Physical examination of 28 joints was done by a single rheumatologist who was expert in bimanual examination of joint. Disease Activity Score- 28 (DAS-28) was obtained using DAS 28 ESR calculator.

The blood samples were taken at 8 AM after 12 h of overnight fasting. The blood samples were centrifuged and the sera were used for biochemical measurements. There were no changes in all participants’ regular diets during past 7 days. Serum leptin concentration was determined using an enzyme-linked immunosorbent assay (ELISA) (LDNr GmbH, Germany), with an intra-assay coefficient of variation (CV) of 3.7–5.5% and an inter-assay coefficient of variation of 5.8–6.8%. Hs-CRP was assessed using a two-site ELISA (CAN-CRP-4360, Diagnostic Biochem). Intra- and inter-assay CV were 5–15.2% and 7.8–9.9%, respectively. Anti-citrullinated cyclic peptide (CCP) levels were determined by ELISA kit (EA 1505–9601, Euroimmun), which measures human IgG antibody against CCP. Rheumatoid factor (RF) assessed by the latex agglutination method. Erythrocyte sedimentation rate (ESR) was measured through Westergren method. Plasma creatinine was measured by Jaffe method (Pars Azmun kit). Colorimetric method (Pars Azmoon kit) was used to determine blood urea nitrogen (BUN).

### 2.3 Statistical analysis

Baseline characteristics of the participants were expressed as mean ± SD for continuous and percent for categorical variables. T-test and Chi-squared test were used to compare continuous and categorical variables respectively. In the first step, comparisons were done between controls and RA patients. Next, RA patients were divided according to DAS-28 score into high activity and low activity group and the comparisons were done between these groups. Further, to increase the possibility of demonstrating the association of leptin with CRP, leptin quartiles were obtained. Then, values for serum CRP were compared between the obtained leptin groups using one-way ANOVA test. Pearson’s correlation test was employed to study the correlation between leptin and CRP. The correlation was also examined in high and low activity disease groups. Univariable and multivariable regression analyses were undertaken to determine contributors to serum leptin concentration in RA patients and Control group.

Analyses were performed by SPSS software (v.18). Two-sided P-value < 0.05 was considered statistically significant.

## 3 Results

Table 1 shows the primary characteristics of the study population. Both low activity and high activity groups received same regimen of drugs. Prednisolone dose was ≤7.5 mg/day, methotrexate was administered in range of 7.5–15 mg per week, and Hydroxychloroquine 200 mg daily for all of them. None of the RA patients were receiving DMARDs other than methotrexate. Serum ESR and CRP values were significantly higher in high activity group compared to low activity group (Table 1). Serum leptin level was significantly higher in RA patients compared to controls (P < 0.01).

Leptin levels correlated significantly with CRP in healthy controls (r = 0.470; p < 0.05), but there was no significant correlation in patients (r = 0.094; p = 0.42) with either High (r = 0.111; p = 0.495) or low activity(r = 0.78; p = 0.658).

For further evaluating the contributors of serum leptin, univariable and multivariable regression was done. Table 2 and Table 3 show univariable and multivariable regression analyses for control group and RA patients considering leptin as dependent variable. CRP, female sex and BMI were significantly correlated with serum leptin level in control group. Only female sex held its significancy in RA patients.

**Fig. 1:**
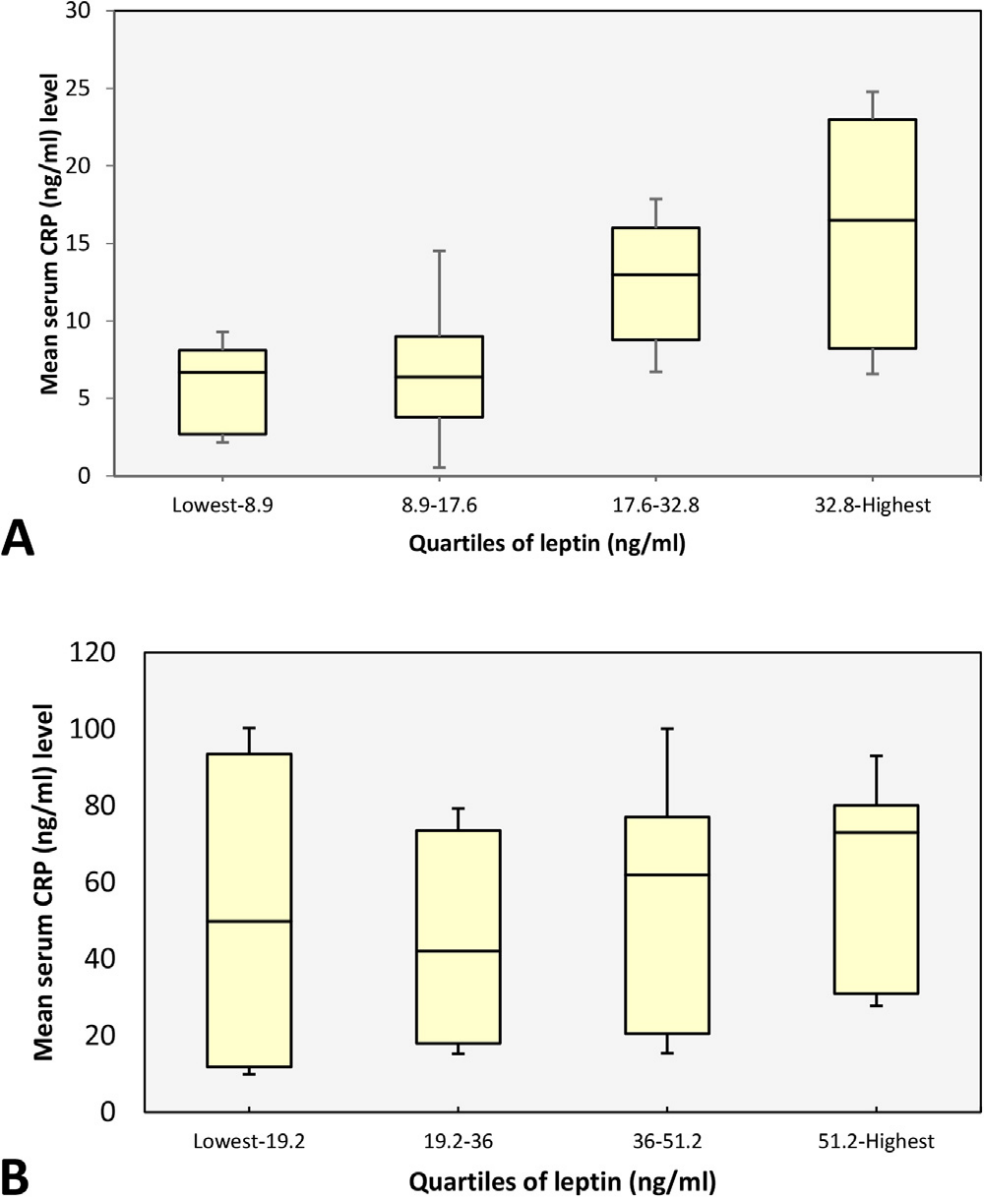
Modified boxplot with error-bars, showing CRP levels in quartiles of leptin. Boxes represent interquartile range (Q1-Q3) and whiskers show error-bars, demonstrating the range of mean ± SD. (A) controls, (B) RA patients. CRP levels are higher in upper leptin quartiles and lower in lower leptin quartiles of healthy control. RA patients do not show such a phenomenon.

We then studied serum CRP levels in different quartiles of leptin using a modified boxplot with error-bars (Fig. 1). Quartiles have been chosen to further investigate the data because of skewed distribution of leptin and CRP. CRP levels were higher in upper leptin quartiles and lower in lower leptin quartiles of healthy control. RA patients did not show such a phenomenon (Fig. 1). Using one-way ANOVA test, no significant difference was observed for CRP between leptin quartiles (P = 0.732) in RA patients.

## 4 Discussion

The main finding of this study was the lost correlation between CRP and leptin in RA patients, which was present in control group. We also observed that leptin was correlated with BMI in controls, this correlation was not present in RA patients.

It is shown that elevated serum levels of pro-inflammatory cytokines such as Interleukins (IL) 1, 2, 6, 8, and tumor necrosis factor (TNF) alpha in chronic inflammatory states can stimulate leptin secretion [15, 21] and leptin itself can increases the production of inflammatory cytokines that are important in pathogenesis of RA [9, 22, 23] and assumably creates a complex cycle connecting metabolism and inflammation [7].

In addition leptin can stimulate the production and may play a role in regulation of CRP in physiological conditions [18]. We previously reported the lost correlation between CRP and leptin in patients with type 2 diabetes mellitus [15], in which inflammation is considered to be a cornerstone of the disease. We also previously showed that in patients with type 2 diabetes, physiological correlations between heat shock protein 70 (HSPA1A) and plasminogen activator inhibitor-1 disappear [17] and pathological correlations between heat shock protein 70 and asymmetric dimethylarginine and also leptin and HSP70 emerge [14, 16]. RA is also a chronic disease with considerable baseline inflammation. In oppose to some studies reporting the correlation between CRP and leptin in RA patients [3, 4, 23], our results show that the correlation is lost in RA patients and the severity of disease does not affect this correlation.

The mechanism by which CRP and leptin are correlated is not fully elucidated. It is assumed that in physiological states leptin can regulate hepatic production of CRP through inflammatory cytokines such as TNF-alpha and IL-1 and they have a positive correlation [15]. On the other hand, in inflammatory states, both leptin and CRP are influenced by different pathways, such as enhanced effect of other inflammatory markers such as TNF-α and IL-1 [15, 23], therefore this physiological correlation disappears.

Some studies in mathematics fields show that the behavior of cytokine network and inflammatory response is very complex and do not obey simple linear equations and behaves non-linear or in other words chaotic [24, 25]. In a chaotic system different pathways and variables can affect the outcome of a process or a disease which can be unpredictable [24, 26]. Many aspects of our findings can be described by chaotic behaviors of inflammatory and immune responses, and inflammation in both diabetes mellitus and RA can be investigated as a chaotic behavior. Influence of different pathways on the markers of inflammation, unpredictable nature of inflammation and the presence of simple triggers such as a small gene defect or an infection in these conditions are some aspects that are in accordance with chaos theory [25, 27]. The chaotic behavior that we mentioned for the inflammatory response can have some steady states also, such as health, sustained inflammation with or without the presence of sustained trigger [27] which is also in accordance to our subject. In our studies, in health we observe some physiologic correlations such as leptin and CRP, but in diseases with sustained inflammation such as RA or diabetes mellitus, we observe loss of some physiologic and emergence of pathologic correlations [14, 15, 16, 17]. One other important aspect of chaotic behavior is the possibility that the inflammation can be controlled by intervening at certain points, especially in the early course of the disease [28]. Future studies on the nature of prolonged inflammation and especially in the field of applying chaos theory into these conditions can be very helpful in controlling them.

Regarding the association of leptin and CRP in RA, the data are contradictory. Yoshino et al. and Otero et al. reported that leptin has positive correlation with CRP and may act as a proinflammatory cytokine in RA [3, 29]. C Popa et al. stated that there is significant inverse correlation between leptin and markers of inflammation such as CRP and suggests that chronic inflammation may lower serum levels of leptin through suppression [23]. Also, there are studies that stated the lack of correlation between leptin and CRP [8, 30] but they just assessed this correlation only in RA patients, and not in healthy subjects and it was just an observation.

The reason of different results among these studies is maybe lack of confounding factors such as sex, percent body fat, smoking, catecholamine and hormonal status that may affect leptin levels. Also, majority of them did not measure CRP levels in control group or did not assess the leptin-CRP correlation in healthy subjects.

The novelty of our study is the comparison of leptin-CRP correlation in RA patients and healthy controls and investigating the reason for this lost correlation.

Serum levels of leptin was significantly higher in RA patients than controls. None of the parameters of disease activity including DAS-28, ESR, and CRP were correlated with serum leptin in RA patients.

There are conflicting data regarding leptin levels in RA. Some of the previous studies reported that serum leptin is elevated in patients with RA compared to healthy controls [2, 11]. In contrast, some other studies showed that serum leptin is similar between RA patients and controls [8, 23] or even lower [31]. Regarding the association of leptin with RA duration and activity, results are also inconclusive. Leptin level was demonstrated to be independent of RA activity or duration in different studies [8, 12, 13, 32]. But, there are also other studies in which, serum leptin was correlated to RA activity and/or duration [2, 4, 5].

Among possible reasons for discrepancy between the above studies, are the different formula for calculating DAS-28 and different cut-offs for grouping patients into low and high activity RA and also lack of considering confounding factors such as sex, age and BMI.

We also observed that the correlation seen between leptin and BMI in healthy subjects is not present in RA patients. Disease activity and gender difference did not affect this observation. Results regarding this association is also contradictory. There are reports stating positive correlation [22, 30, 33], or no correlation [23, 31] between BMI and leptin in RA patients.

In physiological conditions, leptin is mainly regulated by body fat and correlate with BMI [23], but this may not be true in pathologic states such as RA. One reason is besides body mass fat, inflammation is a potent regulator of leptin production [23]. We also assume that this is maybe due to the catabolic nature of pro-inflammatory markers such as IL-1 and TNF-α and also lack of physical activity due to their disability may play a role in the lost correlation between leptin and BMI in RA patients. More studies are required to further investigate this association.

## 5 Conclusion

Serum leptin was elevated in RA patients compared to healthy controls. However, it was not correlated with laboratory and clinical parameters of disease activity or duration. The physiological correlation between BMI and leptin and CRP and leptin was not present in RA patients. According to our knowledge, this is the first study reporting the lost correlation between leptin and CRP and also comparing CRP levels in different leptin quartiles in RA patients and normal population and studying the pattern. We propose that in inflammatory states, some of physiological correlations disappear and pathological correlations may emerge. Prospective studies on this area, especially applying chaos theory, can further clarify the role of inflammation and some other conditions on these correlations and guides us to better understand and maybe control chronic inflammation.

## Declarations

### Author contribution statement

Seyed Reza Najafizadeh: Performed the experiments; Wrote the paper.

Ghasem Farahmand: Performed the experiments; Analyzed and interpreted the data; Wrote the paper.

Ahmad Tahamoli Roudsar: Contributed reagents, materials, analysis tools or data.

Behnam Heidari: Analyzed and interpreted the data; Wrote the paper.

Arash Aghajani Nargesi: Performed the experiments.

Mehrdad Larry: Analyzed and interpreted the data.

Atieh Pajouhi, Alireza Esteghamati: Contributed reagents, materials, analysis tools or data.

Manouchehr Nakhjavani: Conceived and designed the experiments; Contributed reagents, materials, analysis tools or data; Wrote the paper.

### Funding statement

This research did not receive any specific grant from funding agencies in the public, commercial, or not-for-profit sectors.

### Competing interest statement

The authors declare no conflict of interest.

### Additional information

No additional information is available for this paper.

## Figures and Tables

**Table 1:** Primary characteristics of the participants.

	Comparison between patients and controls		Comparison between RA patients	
Variables	RA Patients (n = 75)	Controls (n = 40)	DAS ≤ 3.2 (n =35)	DAS > 3.2 (n = 40)
Age (yr)	47.37 ± 11.81	48.85 ± 10.80	48.09 ± 12.76	46.75 ± 11.04
Female sex (%)	84	85	80	87.5
Weight (kg)	64.41 ± 11.06***	72.30 ± 10.63	63.10 ± 10.41	65.57 ± 11.64
Height (cm)	161.50 ± 7.46***	155.85 ± 6.64	160.48 ± 7.56	162.40 ± 7.36
BMI (kg/m^2^)	24.70 ± 3.98***	29.41 ± 4.56	24.31 ± 3.43	25.04 ± 4.43
Disease duration(yr)	8.83 ± 5.68	––	8.63 ± 6.20	9.00 ± 5.25
DAS-28	3.34 ± 1.21	––	2.31 ± 0.48***	4.25 ± 0.87
Prednisolone (%)†	100	––	100	100
Methotrexate (%)†	100	––	100	100
Other DMARDS	0	––	0	0
Hydroxychloroquine (%)†	100	––	100	100
Urea (mg/dl)	24.83 ± 6.37	24.23 ± 7.98	26.91 ± 5.08 **	23.01 ± 6.86
Cr (mg/dl)	0.85 ± 0.18	1.06 ± 1.15	0.87 ± 0.21	0.81 ± 0.16
Positive RF(%)	87.7	––	80	77.5
Positive anti-CCP(%)	60	––	48.6	70
ESR (mm/h)	23.35 ± 15.95	––	16.20 ± 10.48***	29.60 ± 17.35
CRP (mg/L)	54.35 ± 38.22	10.34 ± 7.41	42.81 ± 30.48*	64.44 ± 41.69
Leptin (ng/ml)	36.78 ± 19.77 **	22.05 ± 16.01	36.29 ± 19.64	37.21 ± 20.14

Data are Mean ± SD; Categorical variables are compared by χ^2^ test, The rest of variables are compared by T-test; † prednisolone dose: <7.5 mg/day, Methotrexate dose: 7.5–15 mg/week, Hydroxychloroquine: 200 mg/day; *P < 0.05,**P < 0.01, ***P < 0.001. BMI, body mass index; DAS-28, Disease Activity Score-28; DMARD, disease-modifying anti-rheumatic drug; RF, rheumatoid factor; anti-CCP, anti-cyclic citrullinated peptides; ESR, erythrocyte sedimentation rate; CRP, C-reactive protein.

**Table 2:** Univariable and multivariable regression analyses for control group considering leptin as dependent variable.

	Univariable		Multivariable	
Variables	Beta	P-value	Beta	P-value
Female sex (yes/no)	0.643	<0.001	0.703	<0.001
Age (yr)	-0.113	0.481	0.06	0.54
CRP	0.47	0.002	0.288	0.01
BMI (kg/m^2^)	0.329	0.035	0.333	0.003

BMI, body mass index. R^2^ for multivariable analysis: 0.69.

**Table 3:** Univariable and multivariable regression analyses for RA patients considering leptin as dependent variable.

	Univariable		Multivariable	
Variables	Beta	P-value	Beta	P-value
Female sex (yes/no)	0.525	<0.001	0.588	<0.001
Age (yr)	-0.106	0.366	-0.081	0.484
BMI (kg/m^2^)	0.039	0.756	0.118	0.318
Disease duration (yr)	0.026	0.822	-0.128	0.286
DAS–28 > 3.2 (yes/no)	0.023	0.843	-0.046	0.74
Positive RF (yes/no)	0.051	0.667	0.013	0.915
Positive anti-CCP (yes/no)	-0.067	0.571	0.111	0.397
ESR (mm/hr)	0.08	0.497	-0.002	0.991
CRP (mg/L)	0.094	0.42	0.064	0.592

BMI, body mass index; DAS-28, Disease Activity Score-28; RF, rheumatoid factor; anti-CCP, anti- cyclic citrullinated peptides; ESR, erythrocyte sedimentation rate; CRP, C-reactive protein; R^2^ for multivariable analysis: 0.372.
